# Preparation and Study of the Antibacterial Applications and Oxidative Stress Induction of Copper Maleamate-Functionalized Mesoporous Silica Nanoparticles

**DOI:** 10.3390/pharmaceutics11010030

**Published:** 2019-01-14

**Authors:** Diana Díaz-García, Perla R. Ardiles, Sanjiv Prashar, Antonio Rodríguez-Diéguez, Paulina L. Páez, Santiago Gómez-Ruiz

**Affiliations:** 1 Departamento de Biología y Geología, Física y Química Inorgánica, ESCET, Universidad Rey Juan Carlos, Calle Tulipán s/n, E-28933 Móstoles (Madrid), Spain; diana.diaz@urjc.es (D.D.-G.); sanjiv.prashar@urjc.es (S.P.); 2 Departamento de Ciencias Farmacéuticas. Facultad de Ciencias Químicas, Universidad Nacional de Córdoba. Ciudad Universitaria, Haya de la Torre y Medina Allende, X5000HUA Córdoba, Argentina; pardiles@fcq.unc.edu.ar; 3 Departamento de Química Inorgánica, Universidad de Granada, Facultad de Ciencias, Campus de Fuentenueva, Avda. Fuentenueva s/n, E-18071 Granada, Spain; antonio5@ugr.es

**Keywords:** mesoporous silica nanoparticles, maleamates, copper, antibacterial, ROS, oxidation reactions

## Abstract

Mesoporous silica nanoparticles (MSNs) are an interesting class of nanomaterials with potential applications in different therapeutic areas and that have been extensively used as drug carriers in different fields of medicine. The present work is focused on the synthesis of MSNs containing a maleamato ligand (MSN-maleamic) and the subsequent coordination of copper(II) ions (MSN-maleamic-Cu) for the exploration of their potential application as antibacterial agents. The Cu-containing nanomaterials have been characterized by different techniques and the preliminary antibacterial effect of the supported maleamato-copper(II) complexes has been tested against two types of bacteria (Gram positive and Gram negative) in different assays to determine the minimum inhibitory concentration (MIC) and minimum bactericidal concentration (MBC). The biological results showed a moderate antibacterial activity against *Escherichia coli* which motivated a more detailed study of the antibacterial mechanism of action of the synthesized maleamate-containing nanosystems and whose findings showed oxidative stress generation in bacterial cells. All the prepared nanomaterials were also tested as catalysts in the “solvent free” selective oxidation of benzyl alcohol, to observe if there is a potential correlation between the catalytic oxidation capacity of the materials and the observed oxidative stress in bacteria. This may help in the future, for a more accurate rational design of antibacterial nanosystems, based on their observed catalytic oxidation activity.

## 1. Introduction

In recent years, mesoporous silica nanoparticles (MSNs) have been extensively used as supports of a wide variety of therapeutic compounds, representing one of the most important support materials in drug-delivery [1,2,3,4]. In most cases, MSNs were loaded with cargo molecules, such as organic-based anticancer drugs, to study their release profile and their potential therapeutic interest [5,6].

However, mesoporous silica-based materials have also been functionalized with metallodrugs of interest in cancer treatment, such as platinum compounds [7,8,9,10,11,12,13,14], titanium compounds [10,15,16,17,18,19,20,21,22,23], tin compounds [22,24,25] and even ruthenium photosensitizers [26], observing promising anticancer activity and a potential future application in clinical trials. The cellular action of these metallodrug-functionalized nanostructured silica-based materials showed that, in most cases, the nanosystems do not release the metallodrug to the biological medium (or the metallodrug release rates are very low), and they usually act as “non-classical” drug-delivery systems, whose cytotoxic activity is due to the action of the entire nanoparticle. Despite the lack of release of the metallodrug to the medium and the fact that the cytotoxic action of these systems is due to the particle action, it seems that the anticancer properties of these materials rely on the triggering of a cell death mechanism which depends highly on the supported metallodrugs (even though they are not released to the medium), as previously reported by our group [21]. 

We decided then to extend our studies of silica-based nanostructured materials functionalized with metallodrugs to different therapeutic applications and distinct metal complexes. The use of new antimicrobials and the confirmation of the importance of the eradication of microorganisms in the first moments of bacterial colonization justify the need to find new therapeutic alternatives associated with the eradication and control of infections. Hypermutability and high bacterial inoculum facilitate the presence of selected multiresistant mutants during antimicrobial treatment. Thus, the general objective of antimicrobial treatment is to minimize the bacterial cell mass, which implies the need to use bactericidal drugs, which do not allow the selection of resistance mechanisms. The lack of effective antimicrobials emphasizes the need for new approaches through the development of novel therapeutic strategies that are capable of eliminating the microorganism by the use of novel nanomaterials.

Mesoporous silica nanoparticles (MSNs) and other mesoporous silica-based materials have usually been studied in antibacterial studies loaded with different antibiotics [27] or other biocides [28]. In addition, mesoporous silicas have also been used as supports for the incorporation of silver or copper nanoparticles [29] and have demonstrated interesting antibacterial properties. However, their use as support of metal complexes with antibacterial properties is still very limited and only a very recent study on the support of nickel and copper Schiff-base complexes with modest antibacterial properties has been reported [30]. Thus, we have used MSNs of particle size below 100 nm to study the incorporation of a maleamic acid fragment, as maleamates are able to perturb the biological action of the enzyme maleamate amidohydrolase [31]. One should take into account that one of the steps in the oxidative degradation of nicotinates in bacteria is the hydrolytic deamination of maleamate to maleate, catalyzed by maleamate amidohydrolase. This might have a negative effect on bacterial viability [32], which might be increased by increasing the concentration of maleamates in bacteria. Thus, we have studied the coordination of copper to the maleamato ligand to obtain supported Cu complexes to determine if there is a synergistic effect from both the copper center and the maleamato ligand that may result in significant antibacterial properties, as it is well known that copper compounds present interesting antimicrobial properties which have been extensively studied in recent years [33,34,35,36,37,38].

In particular, we became interested in copper(II) maleamates, because of the attractive properties that these kinds of compounds have shown in previous antibacterial studies [39,40,41,42,43]. Although these copper(II) compounds did not show a good degree of stability in physiologic medium, (even though they incorporated auxiliary bipyridine and/or phenanthroline ligands in their structure), they presented a potent antimicrobial activity which needs to be examined in detail. Thus, we decided to prepare similar systems, but supported on MSNs, to study their antibacterial effect against two types of bacteria (Gram positive and Gram negative) *Escherichia coli* and *Staphylococcus aureus*. In addition, the induction of oxidative stress in the studied bacteria was also tested for the synthesized nanosystems with interesting results that show that the maleamate-supported ligands, as well as the copper complexes, are triggering a mechanism of oxidative stress in both *E. coli* and *S. aureus*.

Finally, bearing in mind that some of the current studies in biomedicine are focused on the potential use of the catalytic properties of some made-to-measure catalytic metallodrugs, which are able to improve the therapeutic effects in different diseases through their catalytic reactivity [44,45,46,47,48]; and that some copper species have already been supported onto MSNs showing interesting biological and/or catalytic properties [49,50,51,52], we have evaluated the potential influence of the catalytic selective oxidation properties of the studied materials in the induction of oxidative stress in bacteria. We have tested the synthesized nanomaterials as oxidation catalysts in “solvent free” oxidation reactions, to try to determine if there is a potential correlation between the catalytic oxidation capacity of the materials in regular organic reactions and the observed oxidative stress in bacteria. The results indicate that, with a thorough future study in this field, a rational design of antibacterial agents may be possible by simply analyzing their catalytic oxidation capacity, prior to the evaluation of their antibacterial activity.

## 2. Materials and Methods

### 2.1. General Remarks on the Synthesis of Materials

All reactions were performed using standard Schlenk tube techniques in an atmosphere of dry nitrogen. Solvents were distilled from the appropriate drying agents and degassed before use. The reagents used in the preparation of the starting material (mesoporous silica nanoparticles (MSN)), such as tetraethyl orthosilicate (TEOS) and hexadecyltrimethylammonium bromide (CTAB), were purchased from Sigma Aldrich (Tres Cantos, Spain) and Acros Organics (Geel, Belgium), respectively. Triethoxysilylpropylmaleamic acid (maleamic) used in the synthesis of MSN-maleamic was purchased from Fluorochem (Derbyshire, UK). Copper(II) nitrate trihydrate and 5,5’-dimethyl-2,2’-bipyridine used in the preparation of the final material (MSN-maleamic-Cu), were purchased from Scharlab (Barcelona, Spain) and Fluorochem (Derbyshire, UK), respectively. All reagents were used directly without further purification.

### 2.2. General Remarks on the Characterization of Materials

^13^C-CP MAS spectra, were recorded on a Varian-Infinity Plus Spectrometer (Varian Inc., Palo Alto, CA, USA) at 400 MHz operating at 100.52 MHz proton frequency (4 μs 90° pulse, 4000 transients, spinning speed of 6 MHz, contact time 3 ms, pulse delay 1.5 s). X-ray diffraction (XRD) pattern of the systems were obtained on a Philips Diffractometer model PW3040/00 X’Pert MPD/MRD (Philips, Eindhoven, The Netherlands) at 45 kV and 40 mA, using a wavelength Cu Kα (λ = 1.5418 Å). Cu wt % determinations by X-ray fluorescence were carried out with an X-ray fluorescence spectrophotometer Philips MagiX (Philips, Eindhoven, The Netherlands) with an X-ray source of 1 kW and a Rh anode using a helium atmosphere. Thermogravimetry analysis were obtained on a Shimadzu mod. DSC-50Q (Shimadzu, Kioto, Japan) operating between 30 and 950 °C (ramp 20 °C/min) at an intensity of 50 A. N_2_ gas adsorption–desorption isotherms were performed using a Micromeritics ASAP 2020 analyzer (Micromeritics, Norcross, GA, USA). Conventional transmission electron microscopy (TEM) was carried out on a JEOL JEM 1010 (Jeol Ltd., Akishima, Japan), operating at 100 kV using copper grids. For the preparation of the samples, suspensions in ethanol were sonicated for 10 min, subsequently, a single drop of the suspensions was added to the grid, and the solvent was evaporated at room temperature.

### 2.3. Synthesis of Mesoporous Silica Nanoparticles (MSN Material)

The synthesis of MSN material was carried out from a modification of the experimental procedure reported by Zhao et al. [44]. An aqueous solution of CTAB (1 g, 2.74 mmol) was prepared in 480 mL of nanopure water. Sodium hydroxide (2.0 M, 3.5 mL) was added to the solution and the temperature adjusted to 80 °C. The silicon source TEOS (5 mL, 22.4 mmol) was added dropwise under vigorous stirring, and the solution was allowed to react for 2 h. The white precipitate was isolated by filtration and washed with abundant nanopure water and methanol and subsequently dried for 24 h at 80 °C in a stove. Finally, a calcination process was carried out at 500 °C for 24 h.

### 2.4. Preparation of MSN-Maleamic

First, MSN material (1.0 g) was dehydrated by treatment under vacuum at 80 °C for 24 h. Subsequently, the dehydrated MSN material was suspended in toluene (50 mL) and, a solution of the ligand triethoxysilylpropylmaleamic acid (2.0 g in 30 mL of dry toluene) was added to the mixture and stirred at 110 °C for 48 h. The solution was then filtered, and the solid fraction washed with toluene and diethyl ether. The resulting white powder was dried for 24 h at 80 °C.

### 2.5. Preparation of MSN-Maleamic-Cu

A suspension of MSN-maleamic (0.85 g) in 20 mL of acetonitrile was stirred at room temperature and subsequently, 357 mg (1.48 mmol) of copper(II) nitrate trihydrate and 272.3 mg (1.48 mmol) of 5,’-dimethyl-2,2’-bipyridine was added (theoretical level of 10% Cu/SiO_2_). Subsequently, a solution of sodium hydroxide (5 mL, 2% NaOH w/w) was added and the reaction was stirred at 80 °C for 24 h. The solid product was isolated by centrifugation (6000 rpm, 10 min.) and washed several times with acetonitrile and Milli-Q water for elimination the excess of reagents. The resulting light blue powder was dried for 24 h at 80 °C.

### 2.6. Antibacterial Studies

Minimum inhibitory concentration (MIC) and bactericidal concentration (MBC) were determined for MSN-maleamic and MSN-maleamic-Cu by using the standard microdilution method on Mueller Hinton (MH) broth in accordance with standards established by the Clinical and Laboratory Standards Institute [53]. An overnight culture of *Staphylococcus aureus* ATCC 29213 or *Escherichia coli* ATCC 25922 was diluted to achieve a cell density in the range from 10^5^ to 10^7^ colonies forming units per milliliter (CFU/mL) and incubated for 10 min at 37 °C. Bacterial growth was observed at 18 h of incubation. The lowest concentration of the complex that prevented bacterial growth was considered to be the MIC. Viable bacterial counts were obtained for samples without visible bacterial growth by plating on MH agar plates, followed by aerobic incubation at 37 °C for 18 h. The antimicrobial agent concentration that produces the death of 99.9% of initial inoculum was considered the MBC. 

### 2.7. Oxidative Stress Studies

A bacterial suspension (100 μL) of *Staphylococcus aureus* ATCC 29213 and *Escherichia coli* ATCC 25922 was incubated with 100 μL of the corresponding materials MSN-maleamic and MSN-maleamic-Cu at MIC concentration for 1 h at 37 °C. Subsequently, 20 μL of H_2_-DCFDA 20 μM aqueous solution were added. The fluorescence intensity was measured 30 min later with a spectrofluorometer Biotek Synergy HT with the excitation and emission wavelengths at 480 and 520 nm, respectively. Non-treated bacterial suspensions were used as the control. The experiments were carried out in triplicate.

### 2.8. Catalytic Oxidation of Benzyl alcohol

**Figure 1:**
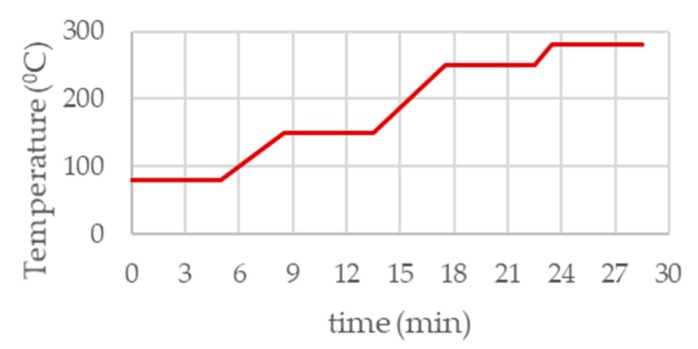
Temperature ramp for gas chromatography (GC) quantification of catalytic products in the oxidation of benzyl alcohol.

MSN-maleamic and MSN-maleamic-Cu were tested as heterogeneous catalysts for benzyl alcohol oxidation under regular catalytic conditions (without light activation) to determine their catalytic activity and selectivity towards the formation of benzaldehyde. In a typical catalytic experiment, 5 mL (48.1 mmol) of benzyl alcohol, 0.5 mL (16.32 mmol) of hydrogen peroxide solution 30% (w/w) in H_2_O, and 25 mg of material were added under nitrogen atmosphere to a Schlenk tube. The mixture was stirred at 110 °C during 6 or 24 h. Subsequently, the reaction mixture was filtered through a nylon filter (0.45 µm) and the filtrate was analyzed by GC-FID (Perkin-Elmer GC Clarus 580, Waltham, MA, USA) with a Velocity^®^ column (dimethylpolysiloxane, 30 m, 0.25 mm, 1.00 µm) to quantify the conversion to benzaldehyde using the temperature program of Figure 1, at an injection temperature of 240 °C and with detector temperature of 300 °C.

## 3. Results and Discussion

### 3.1. Synthesis and Characterization of Maleamic-Functionalized Mesoporous Silica Nanoparticles

MSNs were synthesized following the synthetic method described by Zhao and coworkers [54] with slight modifications on the purification steps as recently reported by our group [26].

**Scheme 1:**
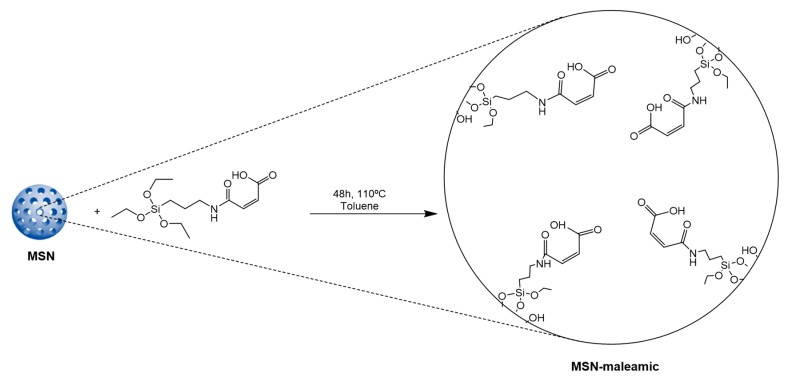
Grafting reaction of MSN material with triethoxysilylpropylmaleamic acid to give mesoporous silica nanoparticles (MSN)-maleamic.

Dehydrated MSN material (treated overnight at 80 °C under vacuum) were functionalized with the maleamato ligand triethoxysilylpropylmaleamic acid (maleamic) to give MSN-maleamic (Scheme 1) by a simple grafting reaction in toluene at mild temperature (80 °C).

**Scheme 2:**
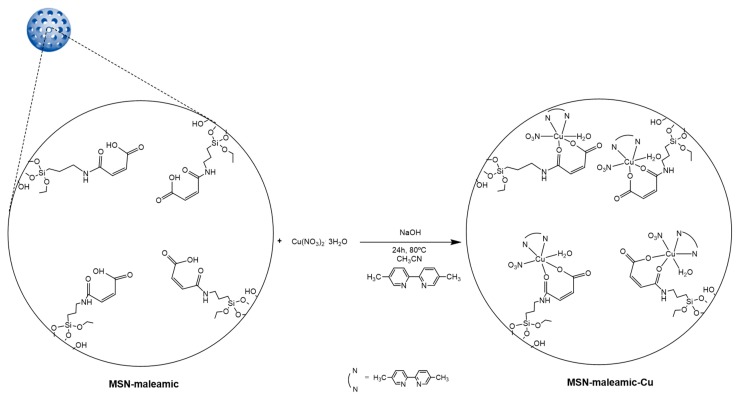
Coordination reaction of mesoporous silica nanoparticles-maleamato ligand MSN-maleamic with copper(II) nitrate in the presence of sodium hydroxide and 5,5’-dimethyl-2,2’-bipyridine to give MSN-maleamic-Cu.

In a subsequent step, the material MSN-maleamic was reacted in acetonitrile with sodium hydroxide and copper(II) nitrate in the presence of 5,5’-dimethyl-2,2’-bipyridine, to coordinate copper centers to the maleamic-supported ligand, giving the copper-functionalized mesoporous silica material MSN-maleamic-Cu (Scheme 2). The theoretical amount of Cu was adjusted to a theoretical level of 10% Cu/SiO_2_. All materials, MSN, MSN-maleamic, and MSN-maleamic-Cu, were characterized by several techniques, such as X-ray diffraction (XRD), X-ray fluorescence (XRF), Fourier Transformed-infrarred spectroscopy (FT-IR), solid-state NMR spectroscopy, diffuse reflectance ultraviolet spectroscopy (DR-UV), and transmission electronic microscopy (TEM).

#### 3.1.1. Quantification of the Functionalization Rate by Thermogravimetry and X-ray Fluorescence

The maleamic-functionalized material MSN-maleamic was characterized by thermogravimetry (TG) to determine the quantity of ligand that had been incorporated in the MSNs. The results showed that, between 120 °C and 650 °C, the material presented a mass loss of ca. 46% (Table 1 and Figure S1 of supplementary material) which corresponds with a functionalization rate of the maleamic ligand of ca. 1.45 mmol of maleamic ligand/gram of MSNs (Table 1). In addition, the TG of the copper-functionalized material showed a similar behavior with a mass loss close to 42% between 120 °C and 650 °C (Figure S2 of supplementary material). Thus, the copper-functionalized material MSN-maleamic-Cu was also characterized by XRF to determine the quantity of supported Cu wt % in MSN-maleamic-Cu. The results showed that the quantity of Cu was of 3.24% (Table 1) which is a slightly higher incorporation rate than that found for other copper complexes supported on mesoporous silica [30] and comparable with previous results obtained by our group when studying the incorporation of metallodrugs of titanium [15,17,18,21,23] or ruthenium [26] in mesoporous silicas. These showed functionalization rates between ca. 1% (for some substituted titanocene derivatives) to ca. 7% (in the case of a grafting of a ruthenium polypyridyl complex). 

The relatively low incorporation of Cu (0.51% wt in the material which is only ca. 32% wt of the starting copper salt) could be due to the difficulty in the deprotonation of the supported maleamic ligand to give the maleamate derivative. This deprotonation reaction was carried out using a diluted solution of NaOH because higher concentrations of NaOH may affect the morphology of the MSNs. Another plausible explanation to the relatively low incorporation of Cu to the material is that a partial saturation of the pores of MSNs with the maleamic ligand limit the coordination of copper centers, thus, decreasing the degree of Cu-functionalization.

#### 3.1.2. Characterization by Powder X-ray Diffraction Studies

**Figure 2:**
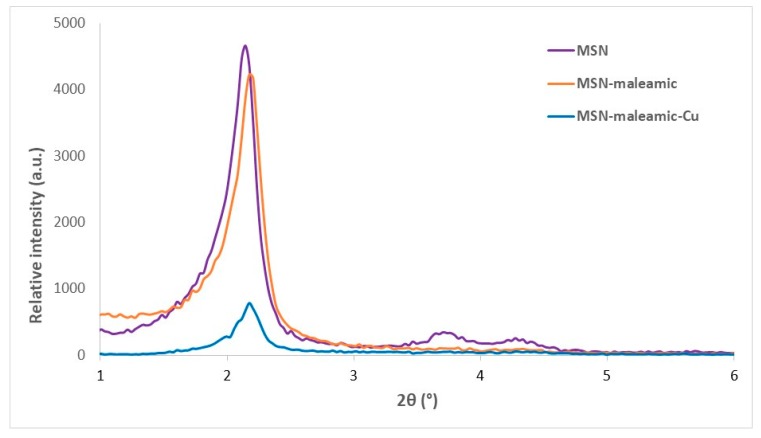
XRD diffractograms of MSN, MSN-maleamic, and MSN-maleamic-Cu.

The starting material MSN and the maleamate-functionalized systems MSN-maleamate and MSN-maleamate-Cu have been characterized by XRD. The diffractogram of the unmodified material MSN (Figure 2) shows three peaks at 2.15°, 3.71°, and 4.28° that correspond to the Miller planes (100), (110), and (200), respectively. The intensity of the peak of the Miller plane (100) is very high while the other two peaks are of low intensity, which is typical behavior for hexagonally ordered mesoporous materials.

It is important to note that, after the first step of functionalization with the maleamic ligand, the intensity of the (100) peak in MSN-maleamic decreases, compared with that observed for the unmodified MSNs. In addition, the position of the peak slightly shifts towards higher 2*θ* angles (Figure 2). The decrease in the intensity is usually associated with the functionalization inside the pores of the mesoporous scaffold, which causes a partial blocking of the dispersion points (pores) of the MSNs. In addition, the change of the position of the diffraction peaks to higher angles is indicative of a pore size decrease. This corresponds to the functionalization with the maleamate ligand mainly inside the pores of the nanoparticles.

Similar changes were observed for MSN-maleamic-Cu, as the diffractogram of this material, after the functionalization with copper, show a decrease in the intensity of the (100) peak in comparison with that of MSN-maleamic. In this case, the position (2*θ*) of the (100) peak in MSN-maleamic-Cu is practically the same as that of MSN-maleamic (Table 2). It is important to note that, after the functionalization steps, the a_0_ parameter decreases (Table 2) as expected for a slight decrease of the pore size.

#### 3.1.3. Nitrogen Adsorption–Desorption Isotherms

**Figure 3:**
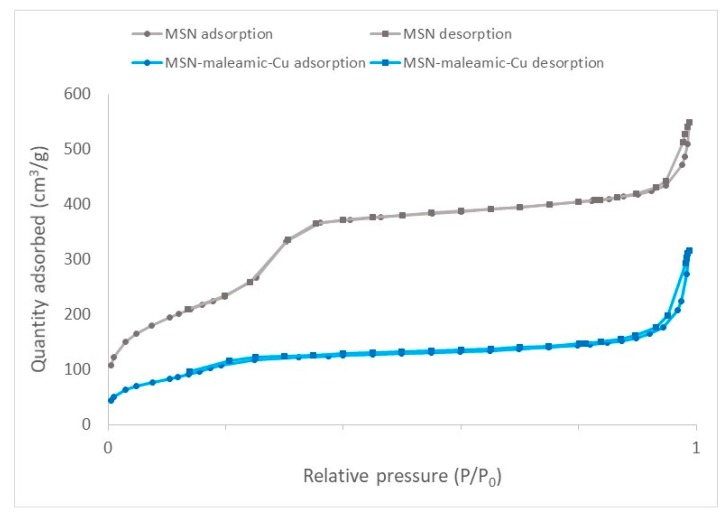
N_2_ adsorption/desorption isotherms of MSN and MSN-maleamic-Cu.

The starting MSN and the copper-modified nanostructured material (MSN-maleamic-Cu) have been characterized by nitrogen adsorption/desorption isotherms. In all cases, one can observe that the isotherms are between type IV and type VI (Figure 3) according to IUPAC classification [55] which are typical of mesoporous silica nanoparticles. The isotherms show a hysteresis loop between *P*/*P*_0_ 1.0 and ca. 0.80 which is usually obtained because of the capillary condensation of nitrogen into the straight pore of the system. Between *P*/*P*_0_ 0.8 and 0.4, the adsorption and desorption behave similarly, however, an additional hysteresis loop (of minimal differences) was observed between *P*/*P*_0_ 0.4 and 0.15, again, probably, due to capillary condensation. 

The physical parameters were obtained after a careful analysis of the Brunauer–Emmett–Teller (BET) isotherms (Table 3). The unmodified MSN material has a BET surface of 853 m^2^·g^−1^, a pore volume of ca. 0.7 cm^3^·g^−1^ and a very narrow distribution of the pore diameter of 3.42 nm. After the functionalization reaction with maleamic ligand and the coordination of copper, the BET surface of MSN-maleamic-Cu is reduced to 414.8 m^2^·g^−1^ and the pore diameter to 3.11 nm, indicating that the functionalization reaction mainly takes place inside the pores of the mesoporous silica nanoparticles.

#### 3.1.4. Solid-State NMR Spectroscopy

**Figure 4:**
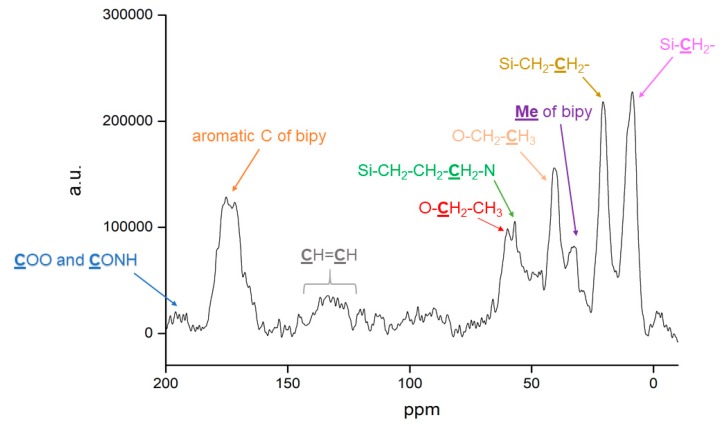
^13^C CP MAS NMR spectra of MSN-maleamic and MSN-maleamic-Cu.

The material MSN-maleamic-Cu has been characterized by ^13^C CP MAS spectroscopy (Figure 4). The spectrum shows the signals corresponding to the carbon atoms of the silylpropylmaleamato moiety, the ethoxide groups, and the bipyridine ligands. Thus, a set of signals of high intensity at ca. 7, 19, and 57 ppm corresponding to the carbon atoms of the Si–CH_2_–CH_2_–CH_2_–N fragment of the ligand is observed. In addition, two signals corresponding to the maleamate moiety are observed, namely, a very broad signal of low intensity between 125 and 140 ppm due to the alkenylic carbon atoms (CH=CH), and a broad signal of very low intensity between ca. 190 and 200 ppm assigned to the carbonyl carbon atoms of the carboxylate (COO^−^) or carboxamide (CONH) groups of the maleamate fragment. Furthermore, the spectrum shows the resonances of the carbon atoms of the bipyridine ligand as a very broad signal at ca. 172 ppm corresponding to the aromatic carbon atoms of the ring and a signal at ca. 37 ppm assigned to the carbon atoms of the methyl groups bound to the aromatic ring). Finally, the spectrum shows the signal of the pendant ethoxide groups at ca. 61 ppm (OCH_2_) and 41 ppm (CH_3_). 

#### 3.1.5. DR-UV and FT-IR Studies

**Figure 5:**
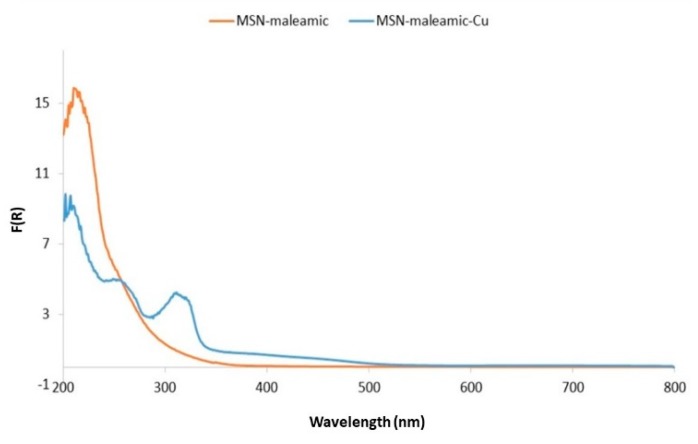
DR-UV spectra of MSN-maleamic and MSN-maleamic-Cu.

The functionalized materials MSN-maleamic and MSN-maleamic-Cu have been characterized by both DR-UV-vis spectroscopy and FT-IR. The DR-UV spectrum of MSN-maleamic (Figure 5) shows an intense absorption peak at 210 nm and a small shoulder of adsorption recorded at ca. 250 nm, which are associated with the maleamic ligand. After incorporation of copper nitrate and the coordination of bipyridine, the spectrum of MSN-maleamic-Cu shows three clear absorption bands at ca. 210, 254, and 309 nm. The first two bands (210 and 254 nm) are due to the maleamato ligand, and the band at 309 is associated with the metal to ligand charge transfer (MLCT) which is usually observed in copper complexes [56]. 

FT-IR spectra of the materials MSN-maleamic and MSN-maleamic-Cu show the typical signals of the mesoporous silica nanoparticles. The spectra present the O–H stretching of the silanol groups and the adsorbed water molecules (between 3500 and 3200 cm^−1^), the siloxane (Si–O–Si) groups (as a broad band at ca. 1100 cm^−1^), the stretching band of the Si–O bonds (at ca. 900 cm^−1^), and the deformation vibrations of adsorbed water molecules at ca. 1625 cm^−1^. 

In addition to the signals of the silica, the appearance of some other bands of very low intensity (associated with both the maleamato ligand and/or the copper nitrate bipyridine fragment) are also observed (See supplementary material Figure S3).

#### 3.1.6. TEM Studies

**Figure 6:**
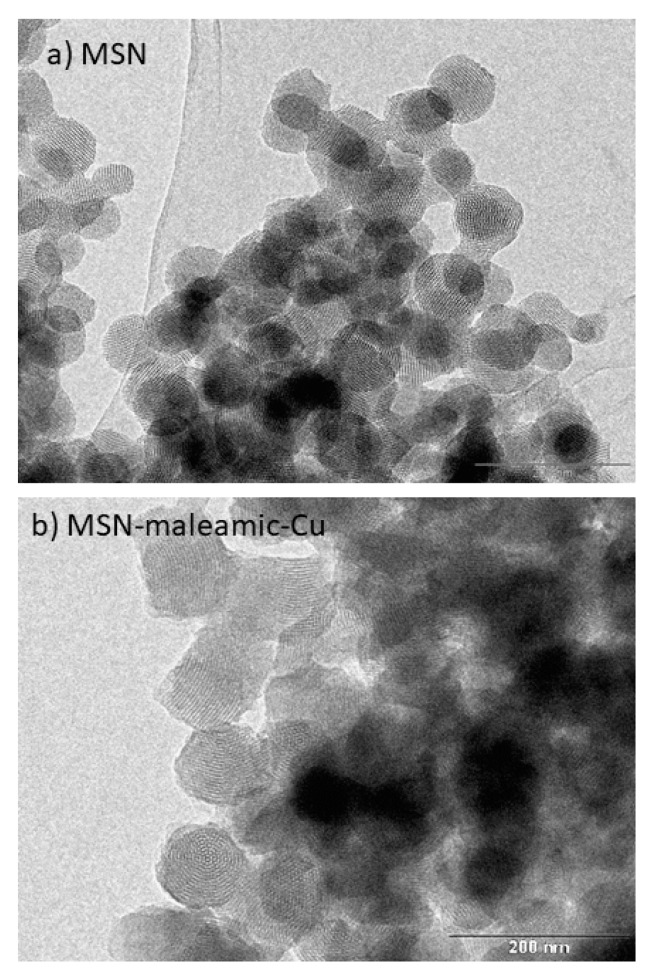
TEM images of MSN and MSN-maleamic-Cu.

Finally, the starting material MSN and the copper-functionalized system MSN-maleamic-Cu, were characterized by TEM. The TEM images (Figure 6a) of the unmodified MSN material show that the nanoparticles are porous spheres with hexagonal ordered porous parallel channels with a narrow particle size distribution (calculated by using ImageJ^®^ program) of ca. 89 ± 3 nm.

After functionalization steps, the TEM images of the nanomaterial MSN-maleamic-Cu show that the morphology of the nanoparticles has not significantly changed observing a similar distribution of the pores and very similar particle size (92 ± 2 nm) than the unmodified MSNs.

### 3.2. Biological Studies of the Synthesized Materials

#### 3.2.1. Antibacterial Tests

The coordination of copper to the maleamato ligand has been studied to obtain supported Cu complexes to determine if there is a synergistic effect from both the copper center and the maleamato ligand that may result in significant antibacterial properties. The MIC and MBC for MSN-maleamic and MSN-maleamic-Cu by using the standard microdilution method on MH broth have been determined. The results (Table 4) showed a low activity of MSN-maleamic and MSN-maleamic-Cu against the Gram positive bacteria *S. aureus*, while a good activity against the Gram negative bacteria *E. coli* was observed. In general, the material MSN-maleamic (with a MIC of 62.5 µg/mL and an MBC of 125 µg/mL) is more active than the material MSN-maleamic-Cu (MIC of 125 µg/mL and an MBC of 125 µg/mL) in *E. coli* in terms of quantity of nanoparticles. However, when one analyzes the MIC referred to the quantity of maleamic fragment in MSN-maleamic and copper centers in MSN-maleamic-Cu, the results show a slightly lower MIC value for MSN-maleamic-Cu (Table 4, data in brackets). The MIC and MBC of copper is similar, if not somewhat lower, than that found for similar systems based on copper nanoparticles, confirming that the bactericidal activity synthesized material MSN-maleamic-Cu in terms of copper MICs (4.1 to >8.1 µg/mL) is comparable, if not slightly higher, than those of other Cu-based nanoparticulated systems [57,58,59,60] which showed MICs of ca. 6 to 20 µg/mL Cu.

When comparing the activity of the studied materials with some clinically used antibiotics, such as ciprofloxacin, erythromycin, gentamicin, tetracycline, doxycycline, chloramphenicol or some others against *S. aureus* populations, the synthesized materials MSN-maleamic and MSN-maleamic-Cu showed a slightly higher activity than commercial antibiotics (with values from 0.5 to 128 µg/mL depending on the antibiotic) [61], while the activity is similar or slightly higher against different *E. coli* populations (with values from 0.5 to >256 µg/mL) [62].

#### 3.2.2. Oxidative Stress Studies

Oxidative stress is usually involved in the toxicity of different antibiotics used in the treatment of bacterial infections [63] which leads to the oxidation of essential macromolecules and induces cell death [64]. Determination of oxidative stress is usually interesting to observe if this is the main reason of the antibacterial activity of the different agents. Thus, we studied the production of reactive oxygen species (ROS) in bacterial suspensions incubated with 100 μL of materials at MIC concentration for 1 h at 37 °C.

**Figure 7:**
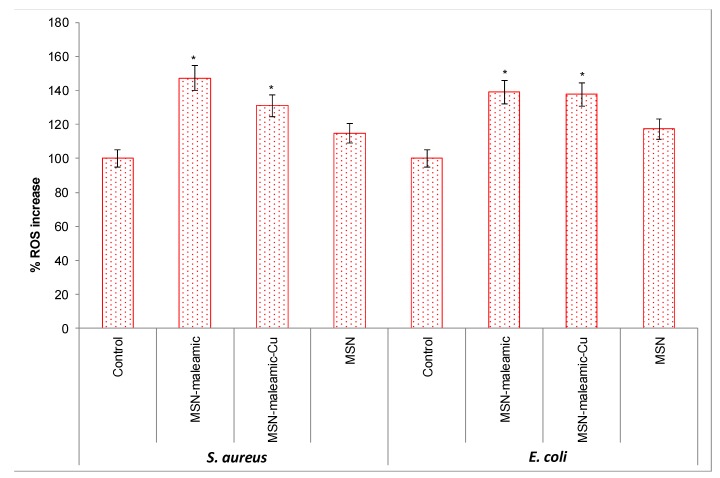
Percentage reactive oxygen species (ROS) increase induced in bacterial suspensions incubated with 100 μL of MSN-maleamic and MSN-maleamic-Cu at minimum inhibitory concentration (MIC) concentrations (the assays were performed at least in triplicate in three independent assays. Data are expressed as means ±SD and analyzed by the Student’s *t*-test. *p* < 0.01 was used as the level of statistical significance compared with control; * = *p* < 0.01).

As can be seen in Figure 7, the synthesized materials MSN-maleamic and MSN-maleamic-Cu generate in *S. aureus* ca. 50 and 30% more ROS, respectively, than untreated bacterial suspensions. In *E. coli*, both materials generate an increment of ROS of 40% respect to control. Interestingly, the material MSN-maleamic seems to generate more ROS than its analogue MSN-maleamic-Cu, which is in agreement with a lower MIC and MBC found in the preliminary antibacterial tests and which may indicate that the mechanism of bacterial death promoted by these kinds of functionalized mesoporous silica-based materials is the oxidative stress generation mediated by an increment of ROS. It is important to note that the major part of the external surface area of MSNs is in the pores of the nanoparticles (higher than 95% of the surface as it corresponds to a mesoporous system), therefore, the major part of the surface in contact with the bacteria is the external part of the pores which are usually the areas with higher functionalization rates. The formation of ROS promoted by unmodified MSN was also studied for both *E. coli* and *S. aureus* observing that the starting material produces much lower quantity of ROS than the functionalized materials, the results observed for unmodified MSN are in agreement with those observed previously in a recent report [65].

It is important to note that, in the synthesized nanostructured systems, the pores are functionalized with either the maleamic ligand or the maleamato-Cu complex and they show influence on the antibacterial behaviour of the materials, even though the bacteria may only be in contact with the external part of the pores. However, in the culture medium, we expect that the nanoparticles may internalize the bacteria and the reactions and formation of ROS may also be possible inside the pores justifying the obtained MICs and ROS production results after functionalization of the materials with the maleamic ligand or the maleamato-Cu complex.

### 3.3. Study of the Catalytic Oxidation Activity of the Synthesized Materials

**Scheme 3:**
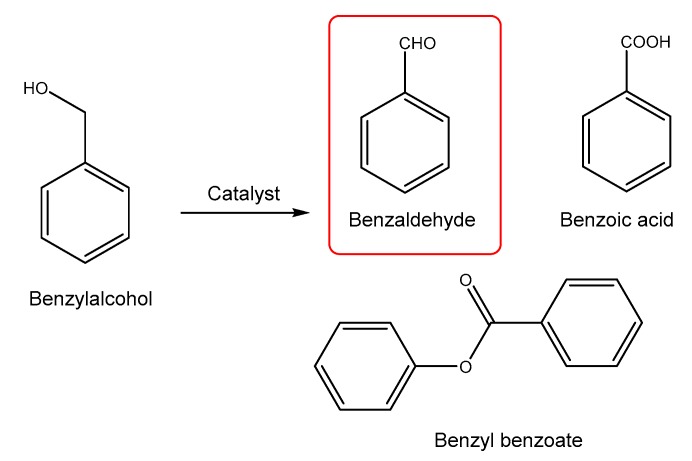
Catalytic oxidation reaction of benzyl alcohol.

A topic of current interest in biomedicine is the potential use of the catalytic properties of some made-to-measure catalytic metallodrugs in therapeutic applications. In view of the results obtained for the generation of ROS in bacteria promoted by MSN-maleamic and MSN-maleamic-Cu nanomaterials, the catalytic activity in a “solvent free” oxidation reaction of benzyl alcohol (Scheme 3) was studied to determine if there is a potential correlation between the catalytic activity of the materials and their production of ROS in bacteria.

Thus, the catalytic activity of MSN-maleamic and MSN-maleamic-Cu in the selective oxidation of benzyl alcohol was tested using H_2_O_2_ as an oxidant agent. Usually, hydrogen peroxide is a more interesting oxidant than organic agents (such as tert-butyl hydroperoxide (TBHP)) in inorganic materials because of the higher mobility of hydrogen peroxide on the surface of the materials, its lower price and its lower environmental impact compared with most of the most widely used organic oxidants. The reactions were carried out at 110 °C during 6 or 24 h, and the results are given in Table 5.

The catalytic study shows that both materials MSN-maleamic and MSN-maleamic-Cu present similar oxidation activity in reactions carried out at 6 h with turnover number (TON) values between ca. 0.6 and 0.9, while the oxidation capacity of MSN-maleamic is much higher at longer times (24 h). In addition, it is important to note that the unmodified MSN was slightly active at 24 h as expected when compared with the activity reported for other mesoporous silica nanostructured materials [66,67]. In addition, the selectivity of the oxidation reaction towards benzaldehyde is very high at 6 h reaction for MSN-maleamic (higher than 90%) while it is much lower in the case of MSN-maleamic-Cu (ca. 75%). The selectivity towards benzaldehyde clearly decreases after 24 h of reaction for both catalysts MSN-maleamic and MSN-maleamic-Cu with a much higher decrease down to ca. 55% in MSN-maleamic. When one compares the total TON (the sum of TON of benzaldehyde and benzyl benzoate) observed in the reactions after 24 h, the results point towards a more efficient oxidation capacity promoted by the maleamic-based material (MSN-maleamic with total TON of ca. 2.4) than its copper analogue (MSN-maleamic-Cu with total TON of ca. 0.9), which is in agreement with the higher ROS production observed and discussed in the previous Section 3.2. In this context, the unmodified MSN has a low total TON of ca. 0.75 after 24 h which is also in agreement with the observation of relatively low ROS in the antibacterial tests. Turnover frequency (TOF) values in h^−1^ are all following the same behavior than TON values as shown in Table 5.

It is noteworthy that the non-expected oxidation behavior of MSN-maleamic-Cu with time, as the total oxidation (total TON) is higher at 6 h (1.08 ± 0.18) than at 24 h (0.87 ± 0.33). We then studied the possible influence of the quantity of hydrogen peroxide in the oxidation capacity of the copper-based material, observing that, in all cases, at higher quantities of hydrogen peroxide in the mixture (more than 0.5 mL hydrogen peroxide in the reaction mixture) the oxidation capacity (TON) is higher at 6 h than at 24 h (See Table S1 of supplementary material). This behavior may be explained by the possibility of Fenton-like degradation processes which may be present at high quantities of hydrogen peroxide as observed previously in several other studies [68,69]. The Fenton-like degradation processes may lead to decomposition of benzaldehyde and/or benzyl benzoate, reducing the apparent total TON of the oxidation reactions. In this context, the degradation processes seem to be more abundant with higher quantities of hydrogen peroxide and after 24 h of reaction. 

## 4. Conclusions

In conclusion, in this preliminary study we have carried out the synthesis and characterization of MSNs functionalized with a maleamato ligand and the subsequent coordination of copper to give an MSN-supported copper maleamate complex. These materials exhibit good activity against *E. coli* and have been shown to generate a high quantity of ROS in the studied bacteria. In addition, our studies on the oxidation of benzyl alcohol catalyzed by the synthesized materials indicates that there seems to be a correlation of the catalytic activity and selectivity in oxidation reactions with the antimicrobial activity and ROS generation, which motivates a more thorough study of a hypothetical structure-dependence in oxidation and antibacterial activities promoted by functionalized mesoporous silica nanoparticles. 

In this context, our future studies will focus, first, on a thorough study of the mechanism of antibacterial action of the materials reported here, determining if they act as “classical” or “non-classical” drug-delivery systems. In addition, our work will also focus on the design and synthesis of new maleamate-functionalized nanomaterials and the incorporation of other metal complexes and ligands to try to clearly determine, in an extensive number of different nanomaterials, if the catalytic activity can be correlated with the potential antibacterial activity. Moreover, the antibacterial studies will extend to other informative bacterial assays, to determine which reactive oxygen species are responsible for the oxidative damage to macromolecules, in addition, we will try to observe if the synthesized materials promote resistance of the bacterial populations. Finally, bacterial DNA fragmentation and membrane potential will also be examined. The results will be compared with different selective catalytic oxidation reactions to support the hypothesis of a relationship between the catalytic activity in oxidation reactions and the antibacterial properties.

## Figures and Tables

**Table 1:** Determination of the quantity of or copper in mesoporous silica nanoparticles-maleamic ligand (MSN-maleamic) or MSN-maleamic-Cu quantified by thermogravimetry or XRF, respectively.

Material	Theoretical Cu wt %	Experimental Cu wt % ^2^	Mass Loss % ^1^	mmol of Maleamic or Cu/g Material
MSN-maleamic	-	-	46.3	1.45 (maleamic) ^1^
MSN-maleamic-Cu	10	3.24	42.1	0.51 (Cu) ^2^

^1^ Determined by thermogravimetry between 120 °C and 650 °C. ^2^ Determined by XRF.

**Table 2:** Powder XRD data of the synthesized compounds.

Material	*hkl*	2*θ* (°)	d*_hkl_* (nm)	a_0_ (nm)
MSN-maleamic	100	2.15	4.099	4.733
	110	3.71	-	-
	200	4.28	-	-
MSN-maleamic	100	2.18	4.053	4.680
MSN-maleamic-Cu	100	2.18	4.023	4.645

**Table 3:** Physical parameters of MSN and MSN-maleamic-Cu measured by N_2_ adsorption–desorption isotherms.

Material	S_BET_ (m^2^/g)	Total Pore Volume (cm^3^/g)	*d_p_* (BJH) ^1^ (nm)
MSN	853.03	0.73	3.42
MSN-maleamic-Cu	414.80	0.32	3.11

^1^ Barrett, Joyner and Halenda.

**Table 4:** Minimum inhibitory concentration (MIC) and bactericidal concentration (MBC) for MSN-maleamic and MSN-maleamic-Cu against *Staphylococcus aureus* and *Escherichia coli*. Data are given in µg/mL of material and data in brackets refer to µg/mL of maleamato ligand or copper centers, respectively.

Material	*Staphylococcus Aureus* ATCC 29213		*Escherichia Coli* ATCC 25922	
		MIC (µg/mL)	MBC (µg/mL)	MIC (µg/mL)	MBC (µg/mL)
MSN-maleamic	250 [41.7]	>250 [>41.7]	62.5 [10.4]	125 [20.9]
MSN-maleamic-Cu	>250 [>8.1]	>250 [>8.1]	125 [4.1]	125 [4.1]

**Table 5:** Turnover number (TON), turnover frequency (TOF), selectivity to benzaldehyde or benzyl benzoate and total TON in the oxidation of benzyl alcohol catalyzed by MSN-maleamic or MSN-maleamic-Cu.

Material	Benzaldehyde			Benzyl Benzoate			Time (h)	Total TON
	TON	TOF (h^−1^)	Selectivity (%)	TON	TOF (h^−1^)	Selectivity (%)		
MSN	0.34 ± 0.28	0.014 ± 0.011	83.7 ± 16.1	0.11 ± 0.04	0.005 ± 0.002	16.3 ± 16.1	24	0.45 ± 0.32
MSN-maleamic	0.69 ± 0.24	0.116 ± 0.041	92.1 ± 1.9	0.06 ± 0.02	0.002 ± 0.001	7.9 ± 1.9	6	0.75 ± 0.26
	1.49 ± 0.34	0.062 ± 0.014	54.2 ± 15.0	0.89 ± 0.21	0.037 ± 0.008	45.8 ± 15.0	24	2.38 ± 0.55
MSN-maleamic Cu	0.85 ± 0.06	0.141 ± 0.010	75.8 ± 10.6	0.23 ± 0.12	0.012 ± 0.001	24.2 ± 10.6	6	1.08 ± 0.18
	0.56 ± 0.14	0.023 ± 0.006	70.1 ± 10.9	0.31 ± 0.19	0.009 ± 0.005	29.9 ± 10.9	24	0.87 ± 0.33

## Supplementary Materials

The following are available online at http://www.mdpi.com/1999-4923/11/1/30/s1, Figure S1: TG of MSN-maleamic; Figure S2: TG of MSN-maleamic-Cu, Figure S3: FT-IR spectra of MSN-maleamic and MSN-maleamic-Cu; Table S1: TON, TOF, selectivity to benzaldehyde or benzyl benzoate in the oxidation of benzyl alcohol catalyzed by MSN-maleamic-Cu with different H_2_O_2_ quantities.^a^
