# Supplementary material for: Preparation and Study of the Antibacterial Applications and Oxidative Stress Induction of Copper Maleamate-Functionalized Mesoporous Silica Nanoparticles
Source: Pharmaceutics. 2019 Jan 14;11(1):30. doi: 10.3390/pharmaceutics11010030 (PMC6359009; doi:10.3390/pharmaceutics11010030)
Supplement: Supplementary file 1 [file pharmaceutics-11-00030-s001.pdf]

pharmaceutics

Supplementary Materials: Preparation and Study of the Antibacterial Applications and Oxidative Stress Induction of Copper Maleamate-Functionalized Mesoporous Silica Nanoparticles

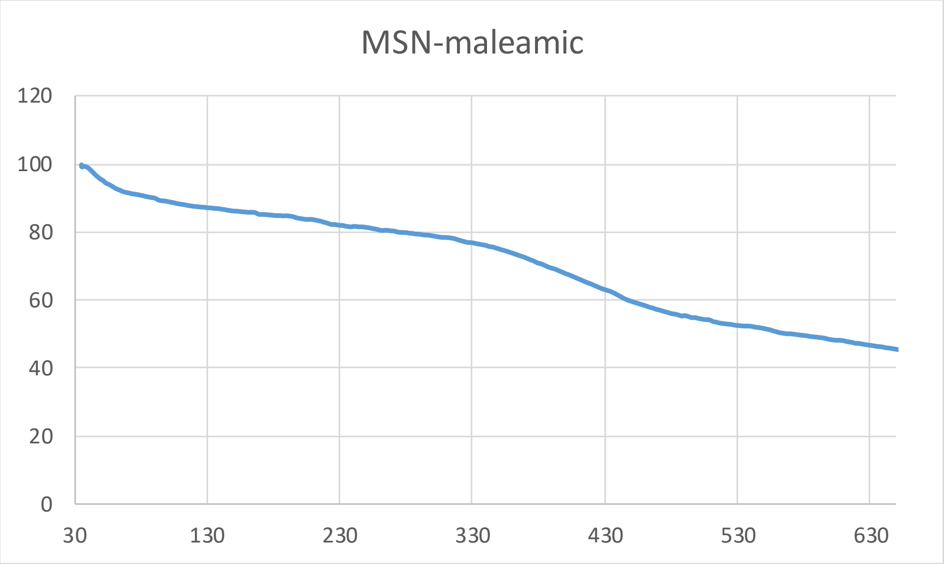

Figure S1. TG of MSN-maleamic.

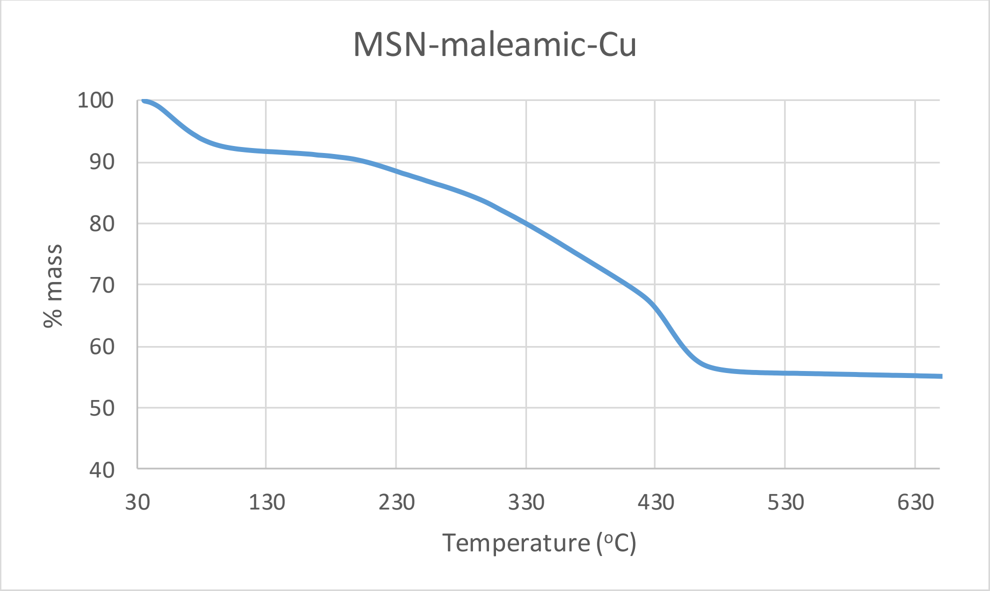

Figure S2. TG of MSN-maleamic-Cu.

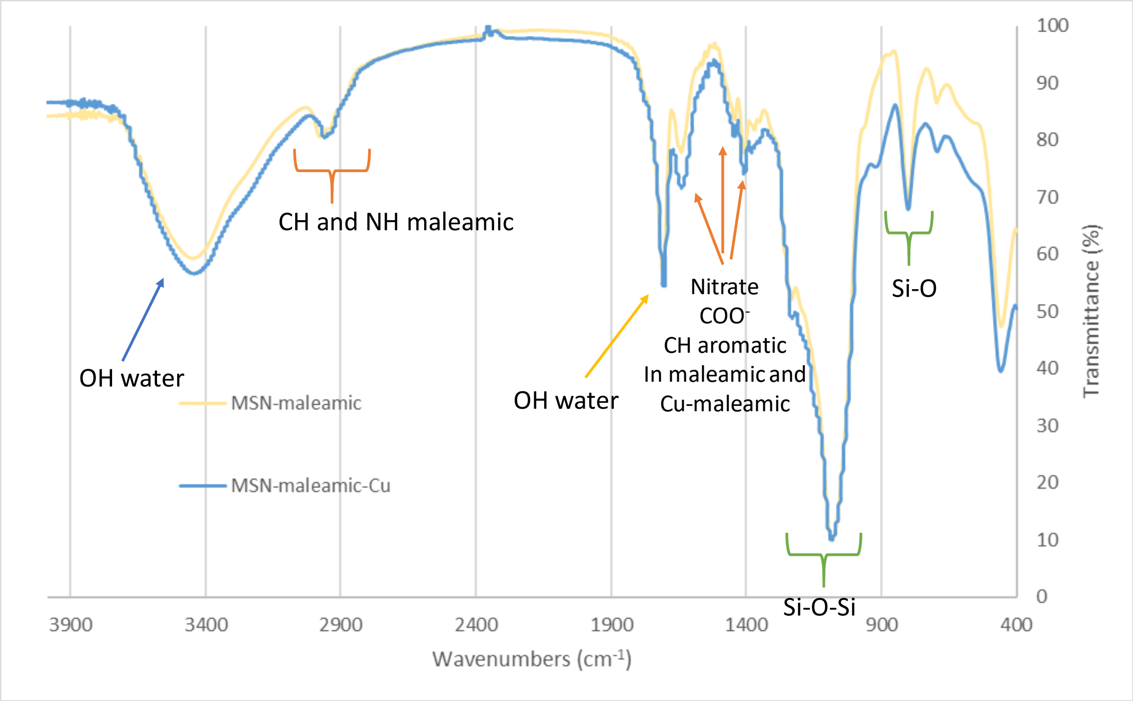

Figure S3. FT-IR spectra of MSN-maleamic and MSN-maleamic-Cu.

Table S1. TON, TOF, selectivity to benzaldehyde or benzyl benzoate in the oxidation of benzyl alcohol catalyzed by MSN-maleamic-Cu with different H_2O_2 quantities.[a]

Material	Benzaldehyde			Benzyl benzoate			Time (h)
	TON	TOF (h^{-1})	Selectivity (%)	TON	TOF (h^{-1})	Selectivity (%)	
MSN-maleamic-Cu	1.33	0.06	67.19	0.65	0.03	32.81	6
(1.5 mL H_2O_2)	1.15	0.19	70.34	0.49	0.08	29.66	24
MSN-maleamic-Cu	1.25	0.21	73.36	0.46	0.08	26.64	6
(1.0 mL H_2O_2)	1.14	0.05	61.13	0.73	0.03	38.87	24
MSN-maleamic-Cu	1.13±0.03	0.19±0	91.73±0.27	0.10±0.007	0.02±0.001	8.27±0.27	6
(0.25 mL H_2O_2)	1.03±0.20	0.04±0.01	90.85±1.64	0.10±0.007	0.02±0.001	9.15±1.64	24

[a] The results without SD correspond to the mean of a unique set of two experiments.
